# RING box protein-1(RBX1), a key component of SCF E3 ligase, induced multiple myeloma cell drug-resistance though suppressing p27

**DOI:** 10.1080/15384047.2023.2231670

**Published:** 2023-08-28

**Authors:** Enfang Bao, Yu Zhou, Song He, Jie Tang, Yunhua He, Mengyuan Zhu, Chun Cheng, Yuchan Wang

**Affiliations:** a Department of Pathogenic Biology, Medical College, Nantong University, Nantong, Jiangsu Province, People’s Republic of China; b Department of Pathology, Affiliated Cancer Hospital of Nantong University, Nantong, Jiangsu, People’s Republic of China; c Department of Pathology, Liyang People’s Hospital, Liyang, Jiangsu, China; d Department of Oncology, Nantong Tongzhou People’s Hospital, Nantong, Jiangsu Province, People’s Republic of China

**Keywords:** RING box-1, p27, multiple myeloma, drug resistance, bone marrow, F-box protein

## Abstract

Multiple myeloma (MM) is a clonal disease of plasma cells that remains, for the most part, incurable despite the advent of several novel therapeutics. The elevated expression of p27 and its association with cell-cycle arrest is speculated to be one of the major mechanisms by which MM cells escape the cytotoxic effects of therapeutic agents. In this study, we demonstrated that RBX1 silencing could inhibit MM cell growth and promote cell drug resistance. RBX1 directly interacted with and triggered the ubiquitination and degradation of p27, ultimately causing p27 reduction. Additionally, cell growth and apoptosis analysis indicated that the role of RBX1 in regulating myeloma cell proliferation and drug resistance resulted from p27 accumulation, which occurred in a Thr187 phosphorylation-dependent manner. Furthermore, the cell-cycle analysis demonstrated that RBX1 overexpression induced cells to enter the cell cycle (S-phase) and partially inhibited chemotherapeutic drugs-mediated cell cycle arrest. Notably, the forced expression of RBX1 also inhibited the cell adhesion-mediated elevation of p27 and induced the accumulation of adherent cells in apoptosis, especially the proteolytic cleavage of caspase-3. Additionally, RBX1 knockdown significantly inhibited myeloma development in SCID-Hu mice and in a human MM xenotransplant model. Overall, these in vitro and in vivo experiments indicated that the RBX1-p27 axis could be a central molecular mechanism by which RBX1 functions as a tumor promoter and stimulates cell growth in chemotherapeutic drugs treated MM cells.

## Introduction

Multiple myeloma (MM) is a plasma cell malignancy, which remains largely incurable with current therapeutic strategies. However, the molecular basis of MM progression and drug resistance are not completely understood.^1,2^ Mounting evidence now suggests that the direct adhesive interactions between MM cells and the bone marrow (BM) microenvironment, also known as ‘cell adhesion mediated drug resistance’ (CAM-DR), play a critical role in tumor development. This phenomenon is speculated to be a major mechanism by which MM cells escape the cytotoxic effects of therapeutic agents. Mechanistically, the BM microenvironment protects MM cells from chemotherapeutic drugs by secreting soluble cytokines, upregulating resistance genes and affecting the cell cycle.^3,4^

Furthermore, the efficacy of certain anti-cancer agents could be reduced when tumor cells exit the cell cycle. Previous studies report that the cell cycle of MM cells was arrested when MM cells were co-cultured with BM stromal cells.^5^ Notably, cyclin-dependent kinase inhibitor p27 was considered a major candidate for this negative regulation of cell growth. Additionally, it was also significantly related to the induction of the CAM-DR phenotype.^6,7^ However, the mechanism by which the BM stroma regulates signaling pathways that upregulate p27 remains unclear. Recently, several lines of evidence support the hypothesis that the intracellular concentration of p27 is largely modulated through the ubiquitin-proteasome pathway.^8^ The pathway involves the phosphorylation of p27 at threonine 187 (T187) during the G_1_ phase via Cyclin E/A-Cdk2 complexes, which mark p27 for recognition by the E3 ubiquitin ligase Skp1, Cullins and F-box protein (SCF) complex.^9^ Additionally, the RING domain-containing protein RBX1 (RING box protein-1)/ROC1 (regulator of cullins-1) are the largest family of the E3 ligases.^7,10^

Moreover, the core of SCF ubiquitin ligases consists of a complex of RBX1-cullins. RBX1 is a 14 kDa protein with a RING finger domain (C3H2C3) that is vital to ubiquitin ligation. Notably, RBX1 interacts with all seven cullin family members to activate the E3 ubiquitin ligases and likely regulates various cellular processes by promoting the degradation of different protein substrates. In *Drosophila*, Rbx1 is required for cell proliferation and embryo development. In *yeast*, Rbx1 plays an important role in the ubiquitination of the cyclin-dependent kinase inhibitor Sic1 during the G_1_ to S phase cell cycle transition. Moreover, RBX1 was reported to be essential for cancer cell proliferation and survival.^10–12^ Upon^13,14^ RBX1 siRNA silencing, cancer cells sequentially undergo cell cycle arrest, senescence and apoptosis.^12^ Tan M et al reported that mouse Rbx1 loss caused early embryonic lethality owing to the significant accumulation of p27, which suppressed proliferation.^10^ Although RBX1 is the key component of SCF E3 ligase, the physiological function of RBX1 in p27 accumulation and its correlation with cell cycle arrest and resistance to chemotherapeutic drugs in MM cells remains uncharacterized.

These studies have highlighted the role of RBX1 in cell growth and the known dysfunction of the SCF E3 ubiquitin ligases in various cancers; therefore, we hypothesize that RBX1 overexpression is required for the survival of MM cells and the promotion of drug cytotoxicity. In this study, the interaction between RBX1 and p27 was demonstrated to be crucial for p27 ubiquitination and proteasomal degradation, which occurred in a Thr187 phosphorylation-dependent manner. Additionally, the direct activation of the RBX1-p27 axis could be a potential central molecular mechanism by which RBX1 exerts its proapoptotic activity in chemotherapeutics-treated MM cells and adherent myeloma cells. Furthermore, we also demonstrated the efficacy of RBX1 in MM cell proliferation using in vivo models. These findings suggest that targeting RBX1 to reduce p27 protein levels is a potential therapeutic strategy for cancer treatment.

## Results

### RBX1 silencing inhibits MM cell growth and induces MM cell drug-resistance

**Figure 1.**
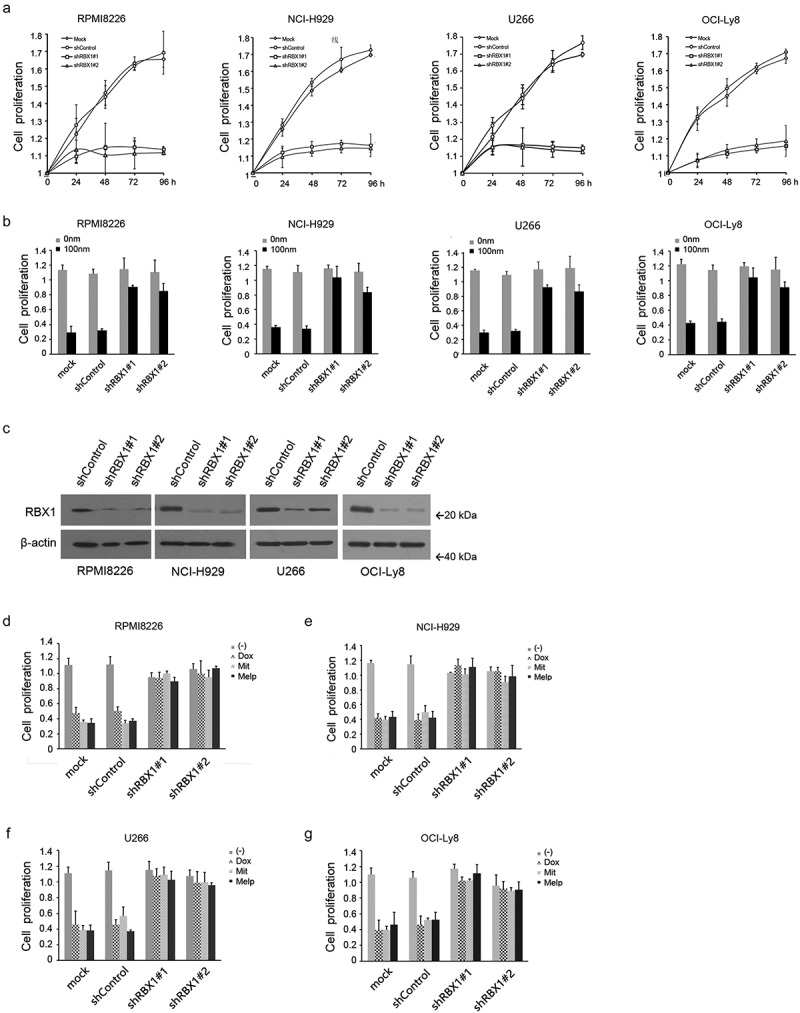
Knockdown of RBX1 inhibits multiple myeloma (MM) cell growth and induces multi-drug resistance. (a) Cell counting kit-8 was used to detect the proliferation of myeloma cell lines RPMI8226, NCI-H929 and lymphoma cell line OCI-Ly8 that were infected with lentivirus-mediated shRBX1 or control shRNA. (b) RBX1 or control shRnas were induced in myeloma cells before treatment with doxorubicin (Dox,100 nM) or DMSO and corresponding cell growth was evaluated. Untreated and without drug treatment cells were used as controls. (c) Cells infected with lentivirus-mediated shRBX1 or shControl were subjected to western blot analyses of RBX1 expression at 72 h post-infection. (d) RBX1 silenced cells were treated with doxorubicin (Dox,100 nM),mitoxantron (Mit, 2 μm) and melphalan (Melp, 10 μm) and the corresponding cell growth was investigated. Each bar represents the mean ± standard error of the mean (S.D) from five independent experiments.

To verify whether RBX1 was dispensable for MM cell proliferation, RBX1 was silenced in three MM cell lines and one lymphoma cell using lentivirus vector-mediated shRNA. The loss of RBX1 inhibited MM cell growth Figure 1(a-c) and proliferation was also inhibited in the four cell lines. To test the hypothesis that MM cells with high RBX1 expression are more drug-resistance and responsible for MM relapse, cells were then grown in the presence or absence of doxorubicin (Dox) at a dose of 100 nM for 48 h. Untreated and shControl-transfected cells with or without Dox served as controls. As shown in Figure 1b, Dox treatment induced significantly less cell death in shRBX1-transfected cells compared with the controls. To further confirm the hypothesis that MM cells with RBX1 suppression are less sensitive to the cytotoxicity of chemotherapeutics, we treated cells with mitoxantrone (2 μM) or melphalan (10 μM) for 48 h. Similarly, shRNA targeting RBX1 induced significantly less growth inhibition and cell death than the shControl transfected controls in the MM cells and lymphoma cell line (Figure 1d–g). Therefore, RBX1 is crucial for MM cell viability, promoting drug cytotoxicity, at least in in vitro MM cell lines.

### RBX1 interacts with p27 and triggers p27 proteasomal degradation

**Figure 2.**
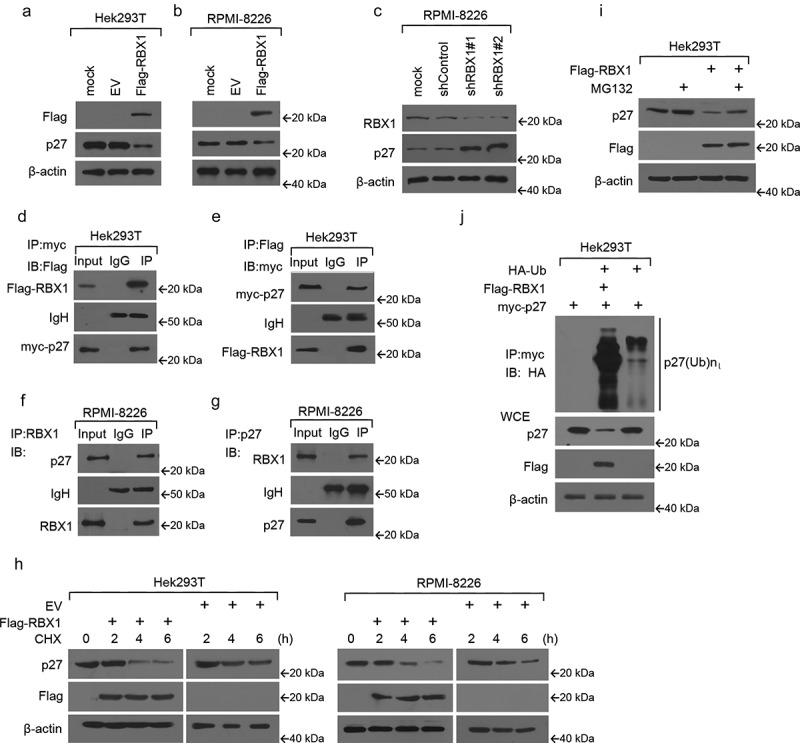
RBX1 interacts with and induces p27 proteasomal degradation in multiple myeloma (MM) cells. (a) Hek293T transfected with pcDNA3-Flag-RBX1 or empty vector (EV) was analyzed using western blot analysis. (b & c) MM cell RPMI-8226 infected with lentivirus-mediated full length RBX1 or empty vector, shRBX1 or shControl, respectively, were then subjected to western blot analyses of RBX1 and p27 expression at 72 h post-infection. (d & e) 293T cells were transfected with pcDNA3-Flag-RBX1 or EV. Proteins in the cellular lysates and immunoprecipitated (IP) proteins were subsequently analyzed using western blot analysis. (f & g) Extracts from RPMI8226 cells were IP with relevant antibodies to RBX1 (f) or p27 (g). Precipitates were then analyzed using immunoblotting. (h) Hek293T and RPMI8226 cells were treated as a or B, and cycloheximide (CHX) was then added for the indicated time. Cell lysates were analyzed using western blotting to detect p27. (i) Hek293T cells were transfected with the indicated plasmids for 48 h, treated with MG132 for 6 h and harvested for the western blotting assay. (j) Hek293T cells were transfected with Flag-tagged RBX1, myc-tagged p27 and a HA-tagged ubiquitin expression construct. WCEs were then IP with anti-myc antibody, and the pellet fraction was analyzed using western blot.

To determine whether the modulation of RBX1 levels had any effect on p27 levels, we transfected Hek293T cells using pcDNA3-Flag-RBX1 and examined the changes in p27 protein levels. As shown in Figure 2a, RBX1 overexpression significantly reduced p27 protein concentration. Cell extracts from RPMI8226 cells transfected with lentivirus-Flag-RBX1 or control lentivirus followed by western blot analysis also showed that increased RBX1 protein concentration significantly decreased p27 levels (Figure 2b). However, RBX1 knockdown by shRNA significantly elevated p27 protein levels (Figure 2c). Following this, Hek293T cells were transfected with differentially tagged expression constructs for RBX1 and p27 and analyzed using immunoprecipitation (IP). As shown in (Figure 2d, e) anti-myc (Flag) antibody downregulated Flag(myc)-tagged RBX1. Moreover, in vivo co-IP experiments revealed endogenous RBX1 complexes with endogenous p27 (Figure 2f, g). As RBX1 interacts with p27 under a physiological condition, we next determined whether reduced p27 protein expression on RBX1 incorporation was a result of impaired protein stability. To this end, we used an inhibitor to eukaryotic translation, cycloheximide (chx), and measured protein half-life using western blotting. We observed that the p27 proteins degraded at a much faster rate when RBX1 was overexpressed, indicating that RBX1 was essential for p27 degradation (Figure 2h). Furthermore, we treated cells with a 26S proteasome inhibitor, MG132, wherein MG132 addition restored p27 protein levels even in the presence of overexpressed RBX1 (Figure 2i). Adding yet more support to this assertion was the observation that RBX1 transfection significantly augmented p27 ubiquitination, which serves as a prelude to protein degradation (Figure 2j).

### RBX1 modulated MM cell drug resistance through the p27-dependent pathway

**Figure 3.**
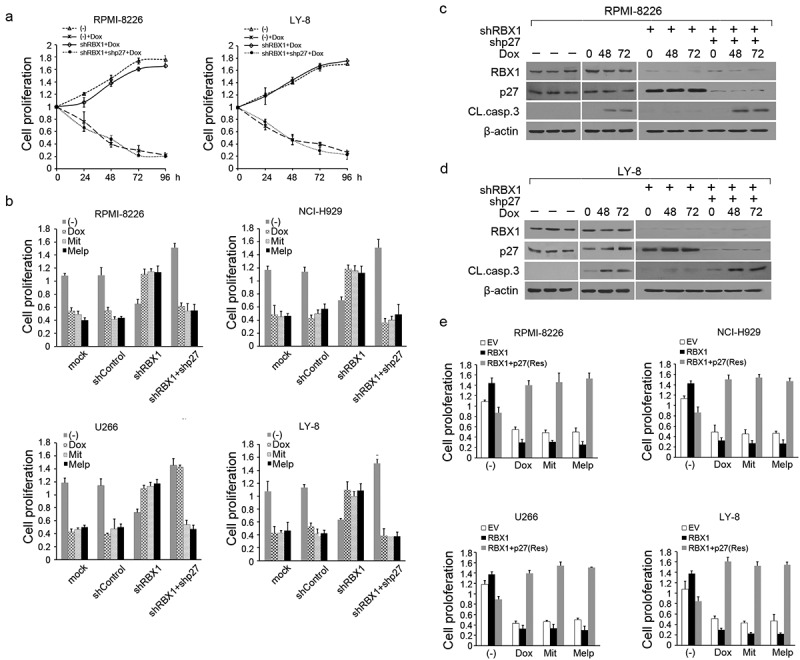
Rescuing p27 attenuates RBX1 induced drug toxic effect in multiple myeloma (MM) cells; (a) RPMI8226 and OCI-Ly8 were infected with lentivirus-mediated shRBX1, shp27 or control shRNA. Then, cells were treated with doxorubicin (Dox, 100 nM) and cell proliferation was assessed using tCell Counting Kit-8. (b) RBX1, p27 or control shRnas were induced in myeloma cells and lymphoma cells for three days before treatment with doxorubicin(Dox,100 nM), mitoxantrone (Mit, 2 μm) and melphalan (Melp, 10 μm) for 48 h. Cell growth was investigated. (c & d) Immunoblot analysis to visualize the expression of the indicated proteins in extracts from RPMI8226 (c) and OCI-Ly8 cells (d) that were infected with the indicated shRBX1 and shp27 constructs and treated or not treated with Dox. (e) the indicated myeloma and lymphoma lines were infected with lentivirus expressing RBX1 or together with p27(Res) or an empty vector and then treated with 100 nm Dox, 2 μm Mit or 10 μm Melp or with DMSO. Cell growth was investigated. Each bar represents the mean ± standard error of the mean (S.D) from five independent experiments.

In light of the significant effect of RBX1 on the degradation of p27, we next investigated if the silencing of p27 expression contributed to chemotherapeutic resistance induced by RBX1 silencing. As shown in Figure 3a, the knockdown of RBX1 increased the viability of RPMI8226 and LY-8 cells treated with Dox in a time-dependent manner; conversely, a significant reduction in cell proliferation was observed in both RBX1 and p27 knockdown cells. Notably, the double knockdown of RBX1 and p27 did not affect the induction of Dox and the other two chemotherapeutics resistance by RBX1 silencing(Figure 3b). Immunoblot analyses indicated that Dox-induced apoptosis was significantly enhanced in RBX1 and p27, thereby simultaneously silencing MM cells and supporting the hypothesis that RBX1 suppression promoted drug resistance towing to p27 accumulation (Figure 3c, d). To further study the biological importance of RBX1-mediated proteolysis of p27, RBX1 overexpressed MM cells were treated with modified p27 (resistant) that failed to interact with RBX1, thereby preventing proteolysis. As shown in Figure 3e, the forced expression of RBX1 increased chemotherapeutics-induced cell growth inhibition compared with control cells. Notably, chemotherapeutics-induced cell growth inhibition was attenuated when p27^Res^ was expressed in RBX1 transfected cells. These results thus provide direct evidence that RBX1 acts as a positive regulatory factor in MM cell drug resistance in a p27-dependent manner.

### RBX1 mediated p27 degradation via Thr187 phosphorylation

**Figure 4.**
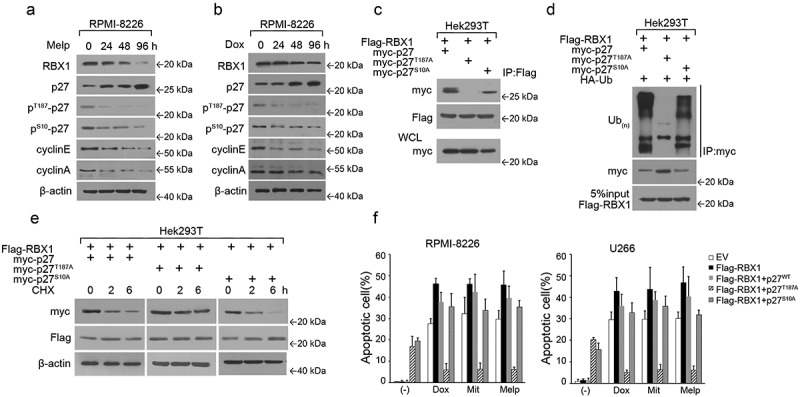
RBX1 mediated p27 degradation that is dependent on Thr187 phosphorylation. (A & B) Immunoblot analysis to visualize the expression of the indicated proteins in extracts from RPMI8226 cells that were treated with Melp (a) and Dox (b). (c) Hek293T cells were co-transfected with myc-p27(WT), p27 (T187A) or p27 (S10A), together with Flag-RBX1 expression vectors. Proteins were precipitated with anti-Flag agarose and analyzed using western blot with myc antibody. (d)hek293t cells were co-transfected with myc-p27 (WT), p27 (T187A) or p27 (S10A), together with Flag-RBX1 expression vectors. WCEs were then immunoprecipitated with anti-myc antibody, and the pellet fraction was analyzed using western blot. (e) Hek293T cells were transfected as in C described, and cycloheximide (CHX) was then added for the indicated time. Cell lysates were analyzed using western blotting to detect p27. (f) RPMI8226 and U266 cells were infected with lentivirus expressing p27 (WT), p27 (T187A) or p27 (S10A) together with RBX1 and then treated with doxorubicin (Dox, 100 nm), Mitoxantrone (Mit, 2 μm) and Melphalan (Melp, 10 μm) or with DMSO. Apoptosis of MM cells was evaluated with a FITC-conjugated Annexin V (Biovision) staining using flow cytometric analysis. Each bar represents the mean ± standard error of the mean (S.D) from five independent experiments.

The serine/threonine phosphorylation of p27 regulates its stability, activity and subcellular localization. The phosphorylation of p27 at Thr187 by the CDK2–cyclin E(A) complexes creates phospho-degrons, which decrease p27 protein levels as a result of ubiquitination and proteasomal degradation.^7,15^ The phosphorylation on Ser10 stabilizes p27 in quiescent cells by maintaining the protein level within the nucleus, where it inhibits CDK2.^8,16^ However, there is no evidence that the phosphorylation of p27 directly control its binding with RBX1. Accordingly, we examined the protein level of RBX1 in MM cells treated with chemotherapeutics and assessed whether the activity of RBX1 is dependent on p27 phosphorylation. Decreased levels of p27 in MM cells treated with chemotherapeutics, while increased levels of RBX1 were observed. As shown in (Figure 4a, b) chemotherapeutic treatments significantly decreased p-T187p27; however, no significant effect was observed on S10p27 protein levels. Furthermore, we also observed that p27^T187A^ did not bind with RBX1 (Figure 4c). To test whether p27 was ubiquitylated directly by RBX1 in a Thr187 phosphorylation-dependent manner, we reconstituted the ubiquitylation reaction of p27 in vivo. The wild type (WT) and S10A p27 were efficiently ubiquitylated when RBX1 was present, but the p27^T187A^ mutant was not (Figure 4d). In keeping with this observation, Thr187 phosphorylation-defective p27 exhibited extended protein stability compared with the WT and S10A p27 when RBX1 was expressed (Figure 4e). We then proceeded to investigate whether these p27 mutations have effects on RBX1-mediated drug resistance in MM cells. Consistent with our earlier observations, chemotherapeutic-induced apoptosis was elevated in RBX1 transfected cells, thus validating that chemotherapeutic resistance in MM cells was attenuated specifically by RBX1 augmentation (Figure 4f). As shown in Figure 4f, constitutive expression of p27^WT^ and p27^S10A^ failed to induce chemo sensitization in RBX1-treated cells. Moreover, only p27^T187A^ expression inhibited apoptosis to a significantly higher extent than either the WT or p27^S10A^. Thus, the direct activation of the RBX1-p27 axis could be a central molecular mechanism by which RBX1 exerts its proapoptotic activity in chemotherapeutics-treated MM cells.

### Effect of RBX1 on MM cell cycle progression

**Figure 5.**
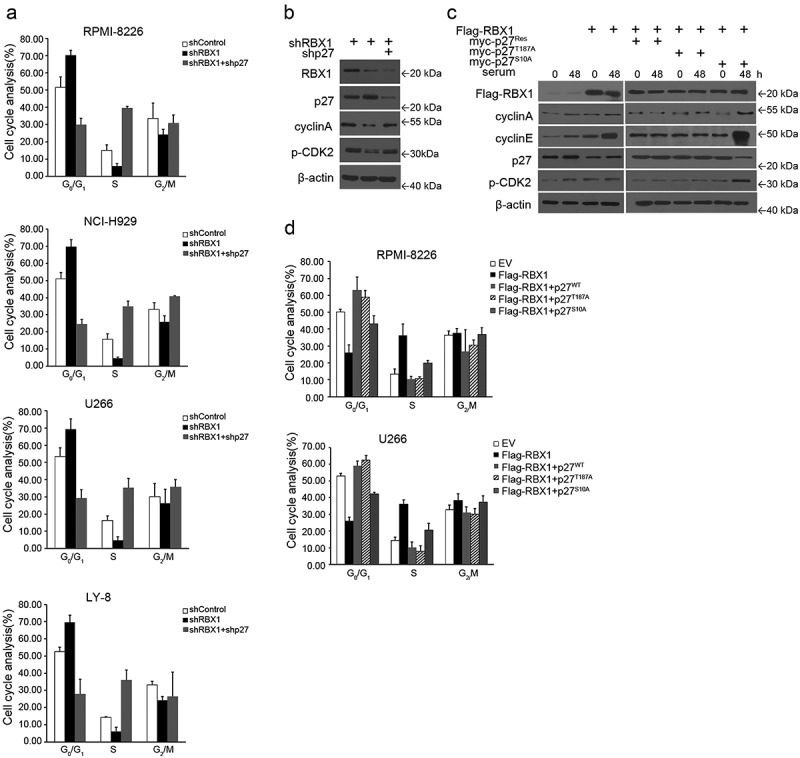
The role of RBX1 in the promotion of cell cycle progression. (a) Myeloma cells and lymphoma cells were infected with lentivirus-mediated shRBX1 or together with shp27. They were then stained with propidium iodide and analyzed using flow cytometry to determine the cell-cycle distribution. (b & c) Immunoblot analysis to visualize the expression of the indicated proteins in extracts from RPMI8226 cells (d) Multiple myeloma (MM) cells infected with lentivirus expressing RBX1 or together with p27 (WT), p27 (T187A) or p27 (S10A) were analyzed using flow cytometry to determine the cell-cycle distribution. Each bar represents the mean ± standard error of the mean (S.D) from five independent experiments.

A drug-resistant phenotype of MM cell is mainly associated with a reversible growth arrest and elevated p27 protein levels.^3,6,17^ To understand the mechanism by which RBX1 regulate MM cell growth, we first investigated the ability of this agent to regulate the cell cycle. The cell cycles of the control cells and cells with RBX1 inhibition or both RBX1 and p27 inhibition were analyzed using flow cytometry. The distributions of cells in the G0/G1, G2/M and S phases were plotted for quantification (Figure 5a). The knockdown of RBX1 in MM cells significantly increased the proportion of cells in the G1 phase, suggesting a role of RBX1 in promoting the cell cycle. However, the double knockdown of RBX1 and p27 impaired cell-cycle arrest induced RBX1 silencing, validating the dependence of RBX1 on p27 (Figure 5a). Furthermore, we investigated if the observed increase in the percentage of cells residing at the G1/S boundary correlated with the decreased expression of the cell-cycle regulator, cyclin A in RBX1 silenced cells. Western blot analysis revealed that cyclinA and phospho-CDK2 protein were significantly decreased when RBX1 was suppressed. Notably, the double knockdown of RBX1 and p27 in MM cells failed to inhibit the expression of cyclin A and phospho-CDK2 (Figure 5b). We, therefore, hypothesized that the simultaneous suppression of RBX1 and p27 in MM cells promotes cell cycle progression, which reduces the chemotherapeutic resistance induced by RBX1 silencing. To further confirm the role of RBX1 in the promotion of cell cycle progression, we transfected MM cells using lentivirus vector-mediated RBX1, or together with p27^WT^ or p27^T187A^ or p27^S10A^mutants. The overexpression of RBX1 led to a decrease in the G_0_/G_1_ population and an increase in the S-phase population (Figure 5d). Additionally, RBX1 overexpression-induced G_0_/G_1_ descent was not observed in p27^Res^ and p27^T187A^ expressed cells, suggesting that the effect of RBX1 was dependent on ubiquitin-proteasome pathways. To elucidate potential mechanisms, we analyzed the expression of a panel of cell cycle regulatory proteins, including several known regulators of G_1_/S progression such as cyclin E, cyclin A and CDK2. As shown in Figure 5c, abundant RBX1 caused the accumulation of cyclin E, cyclin A and p-CDK2, which form a complex to promote the G_0_/G_1_ to S-phase progression. Moreover, these protein levels were also increased in p27^S10A^ combined RBX1 expressing cells, while p27^Res^ and p27^T187A^ treated cells displayed a converse alteration in these protein levels (Figure 5c). Thus, the accumulation of cyclin E, A and activated CDK2 could be responsible for observed G_0_/G_1_ descent in RBX1 overexpressing cells. Thus, these findings suggest that the expression of RBX1 is closely related to the cell cycle of myeloma cells. Moreover, RBX1 could play a regulatory role in the drug-resistant phenotype associated with a cell cycle arrest in a p27-ubiquitylation-dependent manner.

### RBX1 is involved in cell adhesion-mediated p27 accumulation and cell-cycle arrest

**Figure 6.**
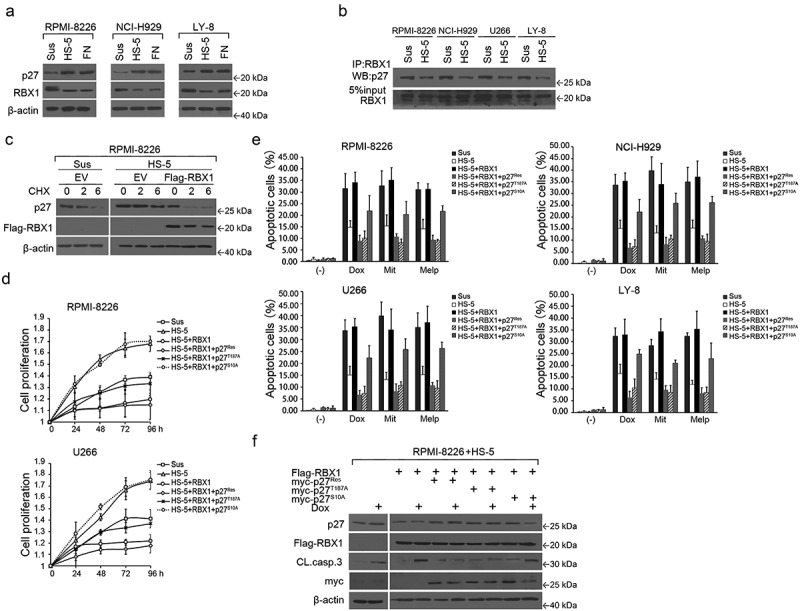
RBX1 regulates p27 degradation in adherent multiple myeloma (MM) cells and reverses CAM-DR. (a) the RBX1 and p27 expression in MM cells and lymphoma cells in suspension (Sus) versus HS-5 or fibronectin (FN) adhesion were analyzed using western blot. (b) Extracts from suspended or adherent MM cells were immunoprecipitated with antibodies to RBX1. Precipitates were then analyzed using immunoblotting. (c) RPMI8226 cells were infected with lentivirus-mediated shRBX1 or control shRna.Then, cells were treated with cycloheximide (100 μM) with and without HS-5 cell adhesion for specific time intervals. p27 protein levels were determined using western blot analysis. (d) MM cells infected with lentivirus expressing RBX1 or together with p27 (WT), p27 (T187A) or p27 (S10A) and were then cultured with HS-5 cells. After 24, 48, 72 and 96 h of culturing, non-adherent cells were removed by gentle washing. The remaining adherent calcein-labeled cells were quantitated in a fluorescence multi-well plate reader using a 494/517 nM filter set. (e) Cells were treated as in C, and then treated with 100 nm Dox, 800 nm Mit or 100 nm Melp or with DMSO for 48 hours in the culture, respectively. Apoptosis of MM cells was evaluated with a FITC-conjugated Annexin V (Biovision) staining using flow cytometric analysis. (f) Immunoblot analysis to visualize the expression of the indicated proteins in extracts from RPMI8226 cells that were infected with the indicated constructs. Each bar represents the mean ± standard error of the mean (S.D) from five independent experiments.

In the BM microenvironment, a proportion of the myeloma cells are insensitive to chemotherapy, which is the main cause of drug resistance and relapse.^1,3,18–21^ It was reported that the cell cycle of MM cells was arrested at the G_0_/G_1_ phase when myeloma cells were co-cultured with BM stromal cells, wherein the main feature of the cells was quiescence.^3,22^ Moreover, p27 accumulation has been associated with the CAM-DR phenotype and could represent a new class of drug-resistance genes.^6^ We, therefore, hypothesized that RBX1 is a likely candidate for regulating p27 ubiquitination and proteasomal degradation in adherent myeloma cells. Accordingly, we examined the levels of RBX1 with or without co-culturing with HS-5 cells and assessed the association with p27 protein levels. A strong negative correlation was observed between p27 and RBX1 levels when myeloma cells were co-cultured with HS-5. The decreased level of RBX1 was also associated with an increased level of p27 in adhered RPMI8226 cells and U266 cells and LY-8 lymphoma cells (Figure 6a). Consistently, p27 demonstrated decreased binding to RBX1 when MM cells were co-cultured with HS-5 (Figure 6b). Notably, the induced expression of RBX1 abolished the cell adhesion-mediated elevation of p27 and shortened its half-life, indicating that RBX1 expression was required for p27 upregulation when myeloma cells were co-cultured with HS-5 (Figure 6c). Additionally, RBX1 was effective in inducing proliferation in adherent MM cells (Figure 6d).

To further determine whether RBX1 induced a protective effect against drug-induced apoptosis, RPMI8226 and U266 cells were cultured with or without HS-5 in the presence or absence of chemotherapeutic drugs. RBX1 overexpression increased the accumulation of adherent cells in apoptosis (Figure 6e), which is associated with the proteolytic cleavage of caspase-3 (Figure 6f). We then sought to determine the importance of p27 phosphorylation in the regulation of cell growth in RBX1 overexpressing cells that were co-cultured with HS-5. Notably, p27^Res^ and p27^T187A^ expression strongly reduced cell viability in RBX1 transfected cells, while p27^S10A^ showed a moderate and gradual decrease in cell viability (Figure 6d). Moreover, the toxic effect of chemotherapeutic drugs was blocked by p27^Res^ or p27^T187A^ expressions in RBX1 transfected cells co-cultured with HS-5. However, p27^S10A^ was not completely refractory to the toxic effect of the chemotherapeutic agents (Figure 6e), which could be attributed to the fact that the S10A forms of p27 were efficiently ubiquitylated when RBX1 was present but the p27^T187A^ mutant was not.^8,9^ Thus, our data indicated that the reduction of RBX1 could represent a predominant mechanism wherein cell-cycle progression is inhibited in adherent myeloma cells via p27 up-regulation.

### RBX1 promotes human MM cell growth in vivo xenograft mouse model

**Figure 7.**
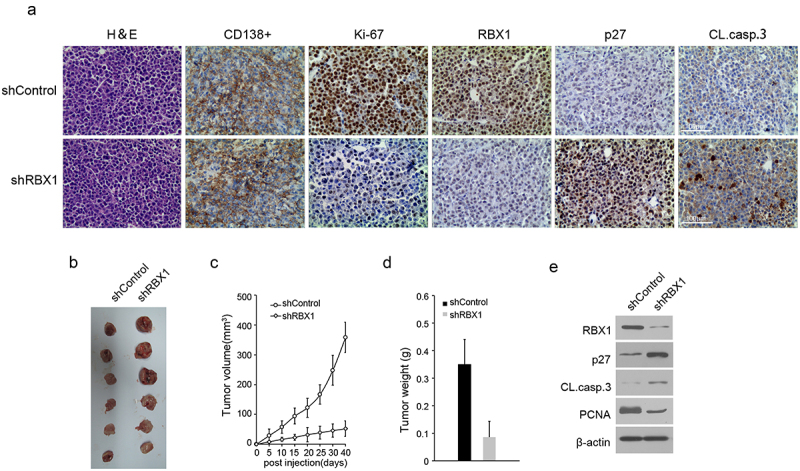
RBX1 silencing suppresses myeloma growth and development in vivo. (a).Histopathology of representative tumors derived from mice to visualize histomorphology (H&E), CD138, RBX1, p27 Ki-67 and spontaneous apoptosis (cleaved caspase 3). Scale bars, 100 μm. (b) Following six weeks of observation, the mice were euthanized and tumor xenografts were removed, weighed and photographed. (c) Summary of the tumor growth curves in nude mice induced by RBX1 silencing and control cells. The average tumor volume and weight (d) were expressed as mean ± standard error of the mean (S.D) in the six inoculated sites for each group. (e) Immunoblot analyses of extracts of the representative tumors derived from the mice in (A).

The tumor-promotive potential of RBX was also evaluated via xenograft tumor formation using human plasmacytoma xenograft and SCID-Hu mouse models.^23^ A subcutaneous visible tumor was observed in the right flank (shControl) in all tested animals during days 8–10 after injection. However, a visible tumor in the left flank (shRBX1) was observed in only two nude mice on day 8. Moreover, the xenograft tumor growth curve showed that the tumor with RBX1 silencing grew much more slowly than the control cells (Figure 7b–d). Six weeks after injection, the tested mice were sacrificed and the tumors were excised for further analysis. The average volume of tumors induced by RBX1 knocked down cells (52 ± 25.9 mm^3)^ was significantly decreased compared with tumors induced by the shControl cells (357.9 ± 50.7 mm^3^, Figure 7c). RBX1 expression in xenograft tumors was analyzed using IHC, wherein RBX1 expression was only detected in tumors induced by shControl cells but little in tumors induced by shRBX1 cells (Figure 7a). The examination of the harvested tumors also showed that RBX1 silencing increased p27 levels and inhibited MM cell proliferation (Figure 7a). Furthermore, RBX1 reduction increased the number of cleaved-caspase-3 positive apoptotic tumor cells compared with the vehicle treatment cells (Figure 7a-e). Overall, these in vitro and in vivo experiments indicated that RBX1 functions as a tumor promoter and stimulates tumor cell growth.

## Discussion

As an essential component of SCF E3 ligase, RBX1 plays a critical role in cancer cell proliferation and survival.^10–12^ However, information on the potential pathological involvement of RBX1 in MM is limited. This study, for the first time, clarifies the functional role of RBX1 in myeloma cell survival and drug resistance by investigating RBX1-induced p27-dependent signaling pathways. Herein, RBX1 silencing in MM cells inhibited cell proliferation and induced multidrug resistance, providing direct evidence for the crucial role of RBX1 in myeloma progression. Additionally, RBX1 was demonstrated to be crucial for regulating p27 ubiquitination and proteasomal degradation. Cell growth and apoptosis analyses indicated that the role of RBX1 in regulating myeloma cell proliferation and drug resistance was dependent on p27 accumulation, especially via Thr187 phosphorylation. The corresponding cell-cycle analysis also demonstrated that RBX1 overexpression induced cells into entering the cell cycle (S-phase), thereby leading to cell-cycle progression. Moreover, RBX1 overexpression partially inhibited chemotherapeutic drugs-mediated cell cycle arrest. Additionally, our experiment showed that cell adhesion-induced p27 accumulation was minimal in RBX1 transfected cells. Furthermore, the induced expression of p27^Res^ or p27^T187A^ was completely refractory to RBX1 overexpression advanced drug toxic effect in adherent MM cells. Thus, these findings reveal the important role of RBX1 in blocking cell adhesion-induced p27 accumulation and cell adhesion-mediated cell arrest, which are the major causes of chemotherapeutic drug resistance.

Myeloma cells are majorly present in the BM, suggesting that the BM microenvironment provides components necessary for the growth and survival of tumor cells.^19,21^ Moreover, a direct cell contact and adhesion between the MM cell and BM could be vital in inducing tumor cell drug resistance.^24,25^ Damiano et al^26^ demonstrated that adhesion protects myeloma cells from Dox and melphalan induced apoptosis. It was also suggested that adhesion to stromal cells protected MM cells from chemotherapeutics-induced apoptosis by causing cell cycle arrest,^5,7^ which is also associated with an increased expression of cell cycle inhibitor p27 and the inhibition of CDK2 activity. However, the molecular mechanism of the stroma regulating the level of p27 and consequently the cell cycle remains unexplored. As a critical cell cycle regulator, p27 arrests cell division and inhibits G_1_/S transition, and cellular p27 levels are largely modulated through the ubiquitin-proteasome pathway.^8,9^ After phosphorylation at Thr187, p27 is recruited to SCF in the nucleus to be polyubiquitinated for degradation through the 26S proteasome.^9,27^ RBX1 is a key component of SCF E3 ligase.^12^ A shRNA library-based functional genomic screen identified *RBX1* as a growth essential gene in various human cell lines but no further mechanistic characterization was performed. Previous studies have revealed that RBX1 is a growth essential gene in yeast, *Caenorhabditis elegans*, *Drosophila*, mouse and several human cancer cell lines.^10,28,29^ In *yeast*, ROC1 is required for the ubiquitination of the cyclin-dependent kinase inhibitor Sic1 during the G1 to S phase cell cycle transition. It was reported that RBX1 silencing significantly inhibited the growth of multiple human cancer cells via the induction of senescence and apoptosis.^29,30^ In the current study, the silencing of RBX1 inhibited MM cell growth and helped to develop drug resistance, which could be rescued by the simultaneous silencing of p27. The mechanistic study also revealed that RBX1 silencing not only led to a significant accumulation of cells at the G_1_/S interphase but also caused a moderate reduction in apoptosis when MM cells were treated with chemotherapeutic agents. However, the possibility that these later changes were the consequence of cell cycle arrest cannot be excluded. Furthermore, chemotherapy induces cell apoptosis by inhibiting the cell cycle and consequently the accumulation of p27 triggered by RBX1 suppression could be associated with this response.

The major finding of this study is the identification of a novel molecular and signaling mechanism that regulates the interaction between MM cells and the BM microenvironment. First, we showed that the RBX1 protein was significantly reduced upon myeloma cell adherence to the stromal cell line HS-5. The overexpression of RBX1 using a lentivirus vector inhibited cell adhesion-dependent p27 elevation and induced myeloma cell proliferation on co-culturing with HS-5. Furthermore, the induced expression of RBX1 in myeloma cells was more sensitive to treatment with specific chemotherapeutic drugs, whereas the introduction of p27^Res^ or p27^T187A^ but not p27^S10A^ completely abrogated the inhibition of myeloma cell death and growth, which was induced by RBX1 overexpression. Thus, it is reasonable to conclude that RBX1 mediates myeloma cell growth and drug resistance through the activity of the ubiquitin ligase.

Furthermore, these data imply that the regulation of RBX1 expression likely plays a significant role in the regulation of MM cell proliferation and chemotherapeutic resistance. RBX1 is also speculated to be an important upstream signaling molecule for the p27 degradation pathway when MM cells adhere to the stromal cells. Clinically, the elimination of myeloma in the BM is an arduous task. The residual myeloma in the BM after therapy is the result of the subpopulations of myeloma cells being rendered resistant to cytotoxic drugs. In conclusion, this study demonstrates that RBX1 plays an important role in MM cell growth and survival, providing direct evidence for the crucial role of RBX1 in myeloma multidrug resistance. Therefore, understanding this molecular pathway is beneficial for the elucidation of this important physiopathological process (myeloma and stroma interaction), which could aid in designing new therapeutic approaches that modify MM cell growth and response to therapy.

## Material and methods

### Cell culture and reagents

Human multiple myeloma cell lines, namely NCI-H929, RPMI-8226 and U266, human lymphoma cell LY-8 and human BMSC line HS-5 were obtained from the American Type Culture Collection (Manassas, VA). All cell lines were maintained in RPMI-1640 medium supplemented with 10% fetal bovine serum. The cells were grown in 5% CO_2_ at 37°C in a humidified atmosphere.

Hek293T was transfected at 70% to 90% confluence, utilizing 2 μl Lipofectamine reagent (Life Technologies) per 1 μg of DNA. Lipofectamine and DNA were incubated in serum-free 1640 or DMEM for 5 min in separate tubes. After incubation, the contents of the two tubes were combined, incubated for an additional 15 min and coated on the cells.

In all assays, MM cells were transfected with shRBX1 or Flag-RBX1 using a lentivirus expression vector system and incubated for 72 h before the initiation of the assay. The efficiency of viral transfection was determined by counting the number of green fluorescent protein (GFP)-expressing cells using flow cytometry, and the transduction efficiency of MM cells was above 95% cells. A total of 1 × 10^6^ cells were treated with mitoxantrone (2 μM) for 48 h. Cell growth was evaluated using a hemacytometer; dead cells were determined using trypan blue staining and the corresponding dead-cell fraction was calculated. Untreated cells and empty-vector (EV) transfected cells with or without drug treatment were used as controls. Similar experiments were performed for Dox (100 nM) and melphalan (10 μM)^4,31^, wherein cells were treated for 48 h.

### Specific gene silencing or overexpression using the lentivirus expression vector system

The lentiviral system in this study was obtained from GENECHEM. This system included the lentiviral vector pGV320 or pGC-LV and two packaging plasmids pHelper 1.0 and pHelper 2.0. Recombinant lentivirus was produced by the transient transfection of 293T cells following a standard protocol.^32,33^ Briefly, the crude virus was concentrated using ultracentrifugation at 10,000 *g* for 90 min. Viral titers were determined by measuring the amount of HIV-1 p24 antigen using an enzyme-linked immunosorbent assay. A 99% transduction efficiency of MM cells was achieved with 3000 ng lentiviral p24 particles/10^6^ cells. The efficiency of viral transfection was determined by counting the number of GFP-expressing cells using an immunofluorescence assay, and the transduction efficiency of MM cells was above 90%.

### Cell proliferation assays

MM cells were split and seeded onto 96-well plates with 2,000 cells per well in triplicates. After 24, 48, 72 and 96 h of culturing, cell proliferation was assessed using Cell Counting Kit-8 (Beyotime, China) following the manufacturer’s instructions.

### Cell cycle analysis

MM cells that were either adhered to HS-5 or kept in suspension for 24–48 h were harvested, washed twice in phosphate-buffered saline (PBS), fixed in cold 70% ethanol for at least 45 min, stained with propidium iodide solution that contains RNaseA for 30 min and finally analyzed using flow cytometry.

### Cell apoptosis assay

The apoptosis of MM cells cultured with Dox or melphalan or mitoxantrone in 96-well plates under different conditions (suspension or coated with HS-5) was evaluated with a FITC-conjugated Annexin V (Biovision) staining using flow cytometric analysis as previously described.

### Adhesion assay

HS-5 cells were plated at a density of 10–20 × 10^3^ per well in black, 24/96-microwell, flat-bottom polystyrene plates and allowed to adhere for 24 h. The MM cell lines RPMI-8226 and U266 (1–2 × 10^5^ were washed thrice with PBS, resuspended in serum-free RPMI medium with 5 μM calcein-blue AM (BD Biosciences) for 30 min at 37°C and 5% CO_2_. The cells were then washed in PBS, resuspended in serum-free medium(SFM) + 1% bovine serum albumin, and plated onto the HS-5 coated plates. After incubation for 2 h, non-adherent cells were removed by gentle washing followed by SFM addition to each well. The remaining adherent calcein-labeled cells were quantitated in a fluorescence multi-well plate reader using a 494/517 nM filter set. Triplicate cultures were set up for every cell population tested.

### Co-IP, p27 degradation assay and immunoblotting

Cells were harvested and rinsed with ice-cold PBS. Ice-cold lysis buffer (150 mM NaCl, 0.5% NP-40, 50 mM Tris (pH 8.0), 2 mM PMSF, complete protease inhibitor cocktail) was added to 1 × 10^7^ cells. Cell lysates were purged 10 times through the 27-gauge syringe, followed by a 30 min incubation with gentle rotation at 4°C. After centrifugation at 4°C for 30 min at 14,000×*g*, the supernatant was used for co-IP. A total of 1 μg of specific antibodies or control IgG (Santa Cruz) was added to the extracted supernatant, shaken for 2 h (4°C), mixed with 30 μl protein A/G (Santa Cruz), incubated for another 2 h (4°C) and washed thrice with washing buffer. Proteins bound to the beads were boiled in 60 μl loading buffer and followed by western blot analysis.

For measurement of p27 degradation, Hek293 with corresponding vector-transfected or myeloma cells in suspension versus HS-5 adhesion were treated with cycloheximide (Sigma), which was added to the culture medium (final concentration: 100 μM, 2 h) to inhibit protein synthesis. At specific time intervals following cell adhesion, cells were lysed and p27 protein levels were determined using western blot analysis to determine p27 protein stability.

The antibodies used for immunodetection were as follows: anti-RBX1 (BD Biosciences, 1:1000), anit-p27 (Santa Cruz, 1:1000),anti-cyclin E (Cell Signaling, 1:1000), anti-cyclin A (Cell Signaling, 1:1000), anti-p-CDK2 (Cell Signaling, 1:1000), anti-β-actin (Sigma, 1:10,000), anti-Flag (Sigma-Aldrich, 1:1000), anti-GFP (Santa Cruz, 1:800) and anti-Myc (Santa Cruz, 1:1000).

### In vivo mouse xenografts assay

All animal experiments were approved by and conformed to the relevant regulatory standards of the Institutional Animal Care and Use Committee at Nantong University. Animals were euthanized when their tumors reached 2 cm or when they exhibited any distress symptoms. SCID-Hu mice were subcutaneously inoculated with U266 cells in 100 ml of serum-free RPMI-1640 medium. Tumor size was measured every 3^rd^ day using a calliper, and tumor volume was calculated using the equation (L×W^2^/2 (where L and W represented the longest longitudinal and transverse diameter, respectively). Following seven weeks of observation, the mice were euthanized and tumor xenografts were removed, weighed and photographed.^32,34^

### Statistical analysis

Data are expressed as mean ± standard error of the mean (S.D). Statistical significance in drug-treated versus control mice in in vitro cultures and tumor xenograft models was determined using the Student’s t-test. Differences were considered significant if *p* < .05. All statistical evaluations were performed using SPSS 13.0.

## Abbreviations

RBX1: RING box protein-1
MM: multiple myeloma
BM: bone marrow
CAM-DR: cell adhesion mediated drug resistance
SCF: Skp1, Cullins, F-box proteins
Dox: doxorubicin
Melp: melphalan
Mito: mitoxantrone
IP: immunoprecipitation.

## Data Availability

All data generated or analyzed during this study are included in this published article.
